# Diabetes Mellitus and Atrial Fibrillation: Mechanistic Insights and Therapeutic Impacts of Glucose-Lowering Drugs

**DOI:** 10.3390/life16010016

**Published:** 2025-12-22

**Authors:** Mihai Grigore, Andreea-Maria Grigore, Martin-Graur Ruxandra-Elena, Verde Ioana, Gabriela Uscoiu, Camelia Nicolae, Ana-Maria Balahura, Adriana-Mihaela Ilieșiu

**Affiliations:** 1 Cardio-Thoracic Department, Carol Davila University of Medicine and Pharmacy, 021021 Bucharest, Romania; mihai.grigore@umfcd.ro (M.G.); ruxandra-elena.martin@drd.umfcd.ro (M.-G.R.-E.); ioana.verde@umfcd.ro (V.I.); gabriela.uscoiu@umfcd.ro (G.U.); camelia.nicolae@umfcd.ro (C.N.); ana-maria.balahura@umfcd.ro (A.-M.B.); adriana.iliesiu@umfcd.ro (A.-M.I.); 2 Internal Medicine and Cardiology Department, “Prof. Th. Burghele” Clinical Hospital, 050653 Bucharest, Romania; 3 Cardiology Department, Colentina Clinical Hospital, 020125 Bucharest, Romania

**Keywords:** diabetes mellitus, atrial fibrillation, glucose-lowering therapies, SGLT2 Inhibitors, GLP-1 receptor agonists, DPP-4 inhibitors, insulin

## Abstract

**Background/Objectives:** Diabetes mellitus (DM) represents a major global public health challenge and is consistently associated with an increased risk of atrial fibrillation (AF). Despite extensive epidemiological evidence linking the two conditions, the underlying mechanisms and the influence of glucose-lowering therapies on AF susceptibility remain incompletely defined. This review aims to summarize the current knowledge on the pathophysiological pathways linking DM and AF and to assess the impact of commonly used antidiabetic therapies on arrhythmic risk. We conducted a narrative review of epidemiological studies, mechanistic research, and cardiovascular outcome trials that evaluate the association between DM and AF. We included data addressing structural, electrical, autonomic, metabolic, and inflammatory mechanisms of AF in diabetes, as well as clinical evidence regarding the impact of metformin, insulin, dipeptidyl peptidase-4 (DPP-4) inhibitors, sodium–glucose cotransporter-2 (SGLT-2) inhibitors, and glucagon-like peptide-1 (GLP-1) receptor agonists on AF incidence or recurrence. **Results:** DM promotes AF development through multiple complementary mechanisms, including atrial fibrosis, electrical conduction abnormalities, autonomic dysfunction, inflammation, glycemic fluctuations, oxidative stress, and expansion of epicardial adipose tissue. These changes create a vulnerable atrial substrate that facilitates both initiation and maintenance of AF. Evidence from recent trials indicates that the arrhythmic effects of glucose-lowering therapies are heterogeneous. Metformin and SGLT-2 inhibitors appear to offer favorable or neutral effects on AF risk. GLP-1 receptor agonists provide substantial cardiovascular benefits, although their specific impact on AF remains under investigation. Insulin therapy has been linked to a higher AF risk, whereas DPP-4 inhibitors show an overall neutral effect with inconsistent findings across studies. **Conclusions:** AF in patients with DM results from complex interactions between metabolic disturbances, structural remodeling, and inflammatory activation. Although several antidiabetic drugs appear to have potential antiarrhythmic effects, further dedicated research is needed to clarify their role in AF prevention and management.

## 1. Introduction

Atrial fibrillation (AF) represents the most frequently sustained cardiac arrhythmia in clinical practice. Its prevalence rises markedly with advancing age, leading to a growing burden of AF in aging populations across the globe [1].

Diabetes mellitus (DM) is one of the most prevalent chronic conditions worldwide and is consistently associated with increased risk of cardiovascular disease, including the development of AF [2]. Although DM independently increases the likelihood of AF, the two conditions share several major risk factors—most notably obesity and arterial hypertension—which further contribute to AF occurrence [3,4,5].

DM is a particularly strong predictor of AF. In a meta-analysis by Huxley et al., individuals with DM exhibited a 40% higher risk of developing AF compared with their non-diabetic counterparts [5]. Moreover, DM, alongside chronic kidney disease, represents one of the most frequent comorbidities that not only predispose individuals to AF but also adversely influence disease progression and overall prognosis [6].

Although the precise mechanisms by which type 2 DM promotes AF remain incompletely understood, it is thought that several interrelated factors contribute to this increased risk. These include altered cardiac energy metabolism, structural remodeling of the myocardium, electrophysiological changes, and autonomic dysfunction arising from diabetic neuropathy [7].

While type 2 DM is recognized as a contributor to the development of AF, the extent to which commonly used glucose-lowering medications may modify arrhythmic risk—either through proarrhythmic or antiarrhythmic effects—remains poorly defined [7].

The present narrative review aims to integrate DM-related structural, electrical, and metabolic alterations within the unifying concept of DM-related atrial myopathy. In addition, we critically appraise the available evidence linking contemporary glucose-lowering therapies to AF risk, with particular emphasis on study design, potential confounding, and clinical interpretability.

## 2. Pathophysiological Pathways Linking AF and DM

Although the association between AF and DM is well established, the underlying pathophysiological pathways remain only partially clarified, reflecting a multifaceted interplay of metabolic, structural, and electrophysiological alterations driven by DM. Chronic hyperglycemia, insulin resistance, and systemic inflammation contribute to cardiac remodeling, fibrosis, autonomic imbalance and others—all of which create a substrate conducive to AF initiation and maintenance [8].

All these mechanisms—including atrial fibrosis, obesity, autonomic dysfunction, and comorbidities—are encompassed within the modern concept of atrial myopathy [9].

### 2.1. Structural Remodeling

One of the principal substrates for AF development in patients with DM is structural remodeling of the atria, particularly left atrial dilation and fibrosis [2]. These alterations arise from a constellation of diabetes-related mechanisms, including heightened oxidative stress, chronic inflammation, and the accumulation of advanced glycation end-products, all of which promote extracellular matrix expansion and fibrotic remodeling [2]. Type 2 DM is also associated with impaired diastolic relaxation, elevated filling pressures, and subsequent left atrial enlargement, favoring the development of AF [10].

Excess epicardial adipose tissue (EAT), common in type 2 DM and obesity, promotes atrial remodeling through mechanical stress, inflammation, and fatty infiltration [11,12]. EAT secretes adipokines and pro-fibrotic factors, generating oxidative stress, apoptosis, and heterogeneous conduction, which facilitate re-entry circuits and AF maintenance [13,14]. Structural and functional atrial alterations, including impaired emptying and fibrosis, further support arrhythmogenesis. Increased EAT thickness is also linked to greater P-wave dispersion, a marker of AF risk and adverse outcomes [15,16]. Overall, EAT expansion creates a substrate that favors both initiation and progression of AF [15].

### 2.2. Electrical Remodeling

DM is strongly associated with an increased susceptibility to atrial arrhythmias, including AF [2]. Much of the mechanistic evidence originates from experimental models, which have demonstrated prolonged interatrial conduction times and alterations in electrophysiological parameters that favor AF initiation and maintenance [2].

These observations are further supported by clinical data. In a study by Chao T-F et al., Atrial Substrate Properties and Outcome of Catheter Ablation in Patients with Paroxysmal Atrial Fibrillation Associated with Diabetes Mellitus or Impaired Fasting Glucose, patients with paroxysmal AF and either DM or impaired fasting glucose who underwent catheter ablation exhibited a higher rate of AF recurrence [17]. Importantly, abnormal glucose metabolism was linked to significant interatrial conduction delay, suggesting that electrical remodeling plays a key role in the arrhythmogenic substrate of diabetic patients [8].

Altered intracellular calcium regulation plays a central role in atrial arrhythmogenesis. In atrial fibrillation, cytosolic Ca^2+^ overload activates calmodulin kinase II (CaMKII), enhances Na^+^/Ca^2+^ exchanger activity, and promotes delayed afterdepolarizations, thereby triggering ectopic impulses and sustaining re-entrant circuits [18]. CaMKII also engages the calcineurin/NFAT pathway, reducing L-type Ca^2+^ currents and shortening the action potential, thereby facilitating AF maintenance [15]. In DM, these mechanisms are further amplified by chronic phase-4 depolarizations, upregulation of small-conductance Ca^2+^-activated K^+^ (SK) and inward rectifier (IK1) channels, and alterations in gap junction proteins such as connexin-40 and connexin-43 [15]. Functional atrial impairments, including reduced passive emptying volume and fraction, contribute additional arrhythmogenic substrate [19].

### 2.3. Autonomic Dysfunction and Glycemic Fluctuations

Autonomic dysfunction is a common complication of DM and represents a form of diabetic neuropathy characterized by an imbalance between sympathetic and parasympathetic activity [2]. This autonomic dysregulation contributes to the development of AF, including asymptomatic or “silent” episodes, by altering atrial electrophysiology and promoting arrhythmogenic conditions. In clinical practice, autonomic dysfunction can be evaluated using heart rate variability, which provides an indirect measure of sympathetic–parasympathetic balance and has been associated with AF susceptibility in DM patients [20].

Glycemic fluctuations have been increasingly implicated in atrial arrhythmogenesis, shifting the focus away from sustained hyperglycemia as the sole glycemic factor associated with atrial fibrillation risk [21]. Interestingly, strict glycemic control does not appear to reduce AF incidence; in contrast, episodes of hypoglycemia can trigger systemic sympathetic activation, thereby increasing the risk of AF [2,22,23,24].

Glycated hemoglobin (HbA1c) has also been investigated as a marker linking DM to AF risk. A meta-analysis of 14 studies involving patients without a prior history of DM found that higher HbA1c levels were associated with an increased risk of AF [25]. Additionally, glycemic variability may promote the production of reactive oxygen species, contributing to oxidative stress and structural and electrical atrial remodeling [5]. Overall, current evidence remains insufficient to demonstrate that intensive glycemic control prevents the development of AF [26].

Effective glycaemic control is recommended as part of comprehensive risk factor management in individuals with DM and AF to reduce burden, recurrence, and progression of AF—indication of class I, level of evidence C [27].

### 2.4. Inflammation

AF is closely linked to obesity and systemic inflammation [5]. Increasing evidence supports the role of inflammatory pathways in the pathogenesis of AF, including atrial structural and electrical remodeling. Chronic inflammation can promote fibrosis, oxidative stress, and autonomic dysregulation, all of which facilitate the initiation and maintenance of AF [5].

Several therapeutic strategies have been proposed to target inflammation and potentially reduce AF risk in DM patients. These include pioglitazone (a thiazolidinedione), polyunsaturated and nitrated fatty acids, vitamins and antioxidants, statins, and DPP-4 inhibitors. While some of these interventions have shown promise in experimental or clinical studies, further research is needed to establish their efficacy in AF prevention specifically among patients with DM [5].

### 2.5. Diabetes-Related Atrial Myopathy: An Integrative Framework

**Figure 1:**
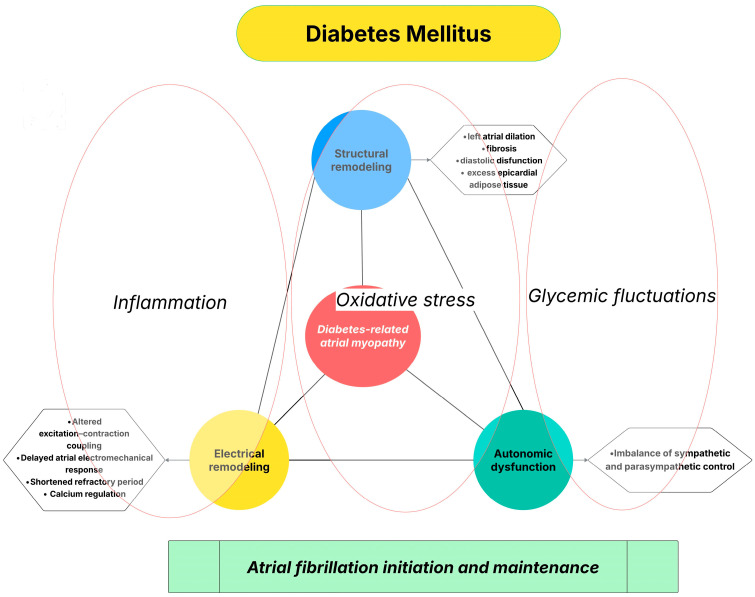
Diabetes-related atrial myopathy as an integrative mechanistic link between DM and AF.

The multiple pathophysiological mechanisms described above—including chronic inflammation, impaired calcium handling, autonomic imbalance, and excess epicardial adipose tissue—do not act in isolation but rather converge to promote a complex atrial cardiomyopathic phenotype in patients with DM (Figure 1). This DM-related atrial myopathy is characterized by progressive structural remodeling, electrical instability, and altered atrial electrophysiology, ultimately creating a substrate highly susceptible to AF.

## 3. Impact of Diabetes on Thromboembolic Risk in AF

AF promotes thrombosis through structural and functional changes, including left atrial dilation, fibrosis, and impaired contraction, which favor blood stasis in the left atrium [28,29]. AF also triggers platelet activation and a hypercoagulable state, with increased fibrin turnover and pro-thrombotic factor expression [30,31,32]. DM amplifies this risk via low-grade inflammation, endothelial dysfunction, insulin resistance, and epicardial fat expansion, all of which enhance platelet reactivity, thrombin generation, and fibrin network density, ultimately increasing stroke risk [33].

While the risk appears broadly similar between type 1 and type 2 DM, some evidence suggests a slightly higher thromboembolic burden in type 2 DM [34,35].

DM is incorporated into the CHA_2_DS_2_-VA score—currently the most extensively validated and widely used tool for estimating thromboembolic risk in patients with AF. The score includes congestive heart failure, hypertension, age ≥ 75 years (doubled), diabetes mellitus, prior stroke/transient ischemic attack or thromboembolism (doubled), vascular disease, and age 65–74 years [3].

## 4. Glucose-Lowering Therapies and AF: Benefits and Risks

Glucose-lowering therapies may influence the atrial substrate and modify the susceptibility to AF. Beyond their metabolic actions, several antidiabetic agents have been shown to mitigate atrial remodeling, reduce inflammation, improve myocardial energetics, or modulate autonomic tone—mechanisms with potential antiarrhythmic relevance [2]. Metformin remains the first-line therapy for type 2 DM and is the most widely prescribed and extensively studied antidiabetic agent worldwide [36].

In recent years, newer antidiabetic classes, SGLT2i and GLP-1RA, have gained substantial attention due to their ability to address multiple cardiovascular risk factors and reduce major cardiovascular events [37,38,39,40]. Contemporary guidelines now recommend SGLT2i and GLP-1RA for patients with diabetes who are at increased cardiovascular risk [41]. Several clinical trials have further explored how these therapies may influence the incidence of new-onset AF, contributing to a growing interest in their potential role in AF prevention [41,42].

It is important to acknowledge that much of the available evidence derives from retrospective cohort studies or post hoc analyses of randomized trials. As such, these associations are inherently susceptible to residual confounding, including channeling bias and confounding by disease severity, whereby patients receiving specific glucose-lowering agents may differ systematically in cardiovascular risk profile, diabetes duration, or comorbidity burden (Table 1).

### 4.1. Traditional Glucose-Lowering Therapies: Metformin, Sulfonylureas, Thiazolidinediones, and Insulin

#### 4.1.1. Metformin

Metformin, a biguanide derivative, is a widely used glucose-lowering drug that reduces hepatic glucose production and enhances glucose uptake by skeletal muscle [43]. It is the most commonly prescribed antidiabetic agent, acting both by inhibiting hepatic gluconeogenesis and by decreasing gastrointestinal glucose absorption [44,45]. Beyond its glycemic effects, metformin has demonstrated cardiovascular benefits, including reductions in blood pressure, left ventricular mass, stroke, heart failure, and cardiovascular mortality [46,47].

Among glucose-lowering drugs, metformin is the most extensively studied for its cardioprotective effects, including reductions in all-cause cardiovascular mortality, stroke, heart failure, and myocardial infarction. However, its potential antiarrhythmic effects remain incompletely defined [48].

In vitro studies have suggested that metformin may protect against atrial arrhythmias by modulating molecular, electrophysiological, and cellular pathways involved in AF [46].

Compared with sulfonylureas, retrospective cohort data indicate that metformin use is associated with a lower risk of developing AF [48].

A large cohort study in Taiwan, including 645,710 patients with DM, reported that metformin was independently associated with a reduced risk of new-onset AF, with a hazard ratio of 0.81 (95% CI 0.76–0.86, *p* < 0.0001). The proposed mechanism involves attenuation of atrial tachycardia-induced myolysis and oxidative stress [47].

The antiarrhythmic effects of metformin are thought to be mediated, at least in part, by activation of AMP-activated protein kinase (AMPK), which facilitates intracellular calcium handling. In patients with long-standing AF, AMPK-related calcium regulation is downregulated. In a study by Deshmukh et al. on the effects of metformin in patients undergoing catheter ablation for AF, DM patients treated with metformin had a lower incidence of atrial arrhythmias [43]. It remains unclear whether this effect is due to direct modulation of atrial electrophysiology via AMPK activation or represents a pleiotropic effect of improved glycemic control [43].

In summary, while randomized controlled trials evaluating metformin’s impact on AF risk are scarce and mostly limited to small, specific populations, for instance post-cardiac surgery patients, observational studies consistently indicate a reduced risk of new-onset AF and AF-related hospitalizations [36].

#### 4.1.2. Sulfonylureas

A higher risk of cardiac arrhythmias, including AF, has been attributed to sulfonylureas (SUs), either through a direct effect on cardiac potassium channels or indirectly via their well-known risk of hypoglycemia, which triggers adrenergic activation. SUs are the second most commonly used antidiabetic drugs, but there is no evidence suggesting they provide protection against AF [49]. Most studies have compared SUs with metformin. A recent retrospective population-based cohort study by Zhou et al., titled Metformin Versus Sulfonylureas for New-Onset Atrial Fibrillation and Stroke in Type 2 Diabetes Mellitus, concluded that patients treated with sulfonylureas had a higher risk of developing AF, particularly those over 65 years of age [49].

In summary, no randomized controlled trials have specifically evaluated the effect of SUs on new-onset AF. Observational studies provide conflicting results, with some suggesting an increased risk compared with metformin, while others report neutral or even lower risk.

#### 4.1.3. Thiazolidinediones

Thiazolidinediones (TZDs) are agonists of the peroxisome proliferator-activated receptor-γ (PPAR-γ) and reduce insulin resistance, representing the only agents directly targeting this mechanism [50]. Although the clinical use of TZDs (pioglitazone, rosiglitazone) is limited due to cardiovascular safety concerns, particularly an increased risk of heart failure, some experimental and clinical evidence suggests that pioglitazone may provide benefits in preventing diabetes-associated AF [41]. Beyond their glucose-lowering effects, TZDs exhibit anti-inflammatory and antioxidant properties, which may influence the development of AF [51]. A comprehensive meta-analysis by Zhang et al., titled Thiazolidinedione Use and Atrial Fibrillation in Diabetic Patients: A Meta-Analysis, including 130,854 patients with diabetes, suggested that TZDs could reduce the risk of AF. Specifically, pioglitazone was associated with a lower risk, whereas evidence for rosiglitazone did not demonstrate a significant reduction [51]. TZD use as second-line therapy was associated with a reduced risk of incident atrial fibrillation compared with other antidiabetic agents [52].

Similarly, a Taiwanese cohort study by Chao et al. reported that TZDs independently decreased the incidence of new-onset AF in patients with type 2 diabetes, with a hazard ratio of 0.69 (95% CI: 0.49–0.91, *p* = 0.028) [5].

In summary, randomized controlled trials have not demonstrated a clear reduction in AF incidence with TZD use, while observational studies consistently suggest that pioglitazone, in particular, may be associated with a lower risk of new-onset and recurrent AF [36].

#### 4.1.4. Insulin

Insulin therapy is typically used in more advanced stages of DM and is frequently associated with suboptimal glycemic control and a higher risk of hypoglycemia compared with other treatments [4]. Recent evidence suggests that glycemic fluctuations, rather than hyperglycemia alone, play an important role in increasing the likelihood of AF [26].

The link between insulin treatment and the higher risk of AF is more complex, since it is typically prescribed to patients with long-standing diabetes and multiple comorbidities, including heart failure. For this reason, establishing a direct causal relationship between insulin therapy and AF risk it is difficult to be proven and remains challenging [53,54].

In summary, no RCTs have specifically assessed the relationship between insulin treatment and new-onset AF, but observational studies suggest a higher risk among insulin-treated patients [36].

### 4.2. Contemporary Glucose-Lowering Therapies: DPP-4 Inhibitors, GLP-1 Receptor Agonists, and SGLT2 Inhibitors

#### 4.2.1. Dipeptidyl Peptidase-4 Inhibitors

Dipeptidyl peptidase-4 inhibitors (DPP-4i) is a newer class of antidiabetic drugs with potential cardiovascular effects [2]. DPP-4i is a transmembrane glycoprotein whose main substrates are GLP-1 and GIP. Despite the anti-inflammatory, anti-fibrotic, and antioxidant effects of DPP-4i which could influence the development of AF, current clinical evidence is limited [5]. In a Taiwanese cohort of 480,000 patients with DM, DPP-4i use in combination with metformin was associated with a lower risk of AF compared with patients not receiving DPP-4i [51]. However, a meta-analysis by Patoulias DI et al. (*Cardiovascular Efficacy of Dipeptidyl-Peptidase-4 Inhibitors*) found that DPP-4i did not significantly modify the risk of AF (RR = 0.95, 95% CI: 0.78–1.17, I^2^ = 0%) [55].

Overall, evidence from Randomized Controlled Trials and observational studies indicates that DPP-4i have a neutral to modest effect on new-onset AF, with some real-world studies suggesting a lower risk in specific populations, while other studies report higher AF incidence compared with SGLT2i [56,57,58,59]. Differences in patient selection, DM duration, and confounding factors likely contribute to these divergent findings, highlighting the need for further investigation [36].

#### 4.2.2. Glucagon-like Peptide-1 Receptor Agonists (GLP-1 RAs)

GLP-1 is an incretin hormone released from enteroendocrine cells in response to nutrient intake, regulating postprandial glucose by enhancing insulin secretion and suppressing glucagon release [60]. GLP-1 RAs are widely used in patients with type 2 DM, primarily for their antihyperglycemic effects, and secondarily for cardiovascular benefits, including reductions in blood pressure and total cholesterol [61]. Importantly, GLP-1 RAs are not associated with significant hypoglycemic episodes [62].

At the myocardial level, GLP-1 RAs improve energy metabolism by decreasing dependence on fatty-acid oxidation and stabilizing glucose availability [63]. They also enhance mitochondrial function, partially through the modulation of the renin–angiotensin–aldosterone system [64]. GLP-1 RAs could influence atrial remodeling by reducing EAT and associated inflammatory signaling, by lowering circulating concentrations of advanced glycation end products and profibrotic markers, and by improving intracellular calcium handling, a critical determinant of atrial electrical stability [65,66].

Large-scale, placebo-controlled cardiovascular outcome trials have demonstrated that GLP-1 RAs confer significant cardiovascular protection, including a 16% reduction in non-fatal stroke among patients with type 2 DM at high cardiovascular risk. Consequently, these agents are strongly recommended in current guidelines for patients with type 2 DM and established atherosclerotic cardiovascular disease [67,68].

Apart from these benefits, their effect on incident AF remains unclear.

Initially, GLP-1 RA were considered to be associated with an increased risk of development of AF [69]. This concern was raised by the Harmony program, which assessed albiglutide and observed a higher number of AF/atrial flutter events in the albiglutide-treated group [61,69].

At the moment there are still insufficient data of GLP-1 RA focusing the AF prevalence [61]. In a review by Hamedi Z et al. [59], summarizing six trials (20,598 patients) with GLP-1 agonists treatment (LEADER—Liraglutide [70], SUSTAIN-6—Semaglutide [71], REWIND- Dulaglutide [72], HARMONY- Albiglutide [69], ELIXA- Lixisenatide [73] and PIONEER-6- Oral Semaglutide [74]), the authors reported similar rates of serious AF events in GLP-1 RA and placebo groups (1.35% vs. 1.37%, respectively [74].

A recent cohort study in Taiwan found no difference in the risk of developing AF between patients treated with GLP-1 RAs and those receiving DPP-4 inhibitors [75].

GLP-1 RAs are known to increase heart rate, although the underlying mechanism is still not fully understood. Two hypotheses have been proposed: direct stimulation of the sinoatrial node, or a compensatory increase in heart rate secondary to the decrease in blood pressure [61,76].

Overall, current evidence does not indicate a consistent connection between GLP-1 RAs therapy and AF development in patients with DM and cardiovascular disease [61,77].

However, a recent meta-analysis including over 3,700 patients with heart failure with preserved or mildly reduced ejection fraction reported a 46% reduction in the incident AF among those receiving GLP-1 RAs, suggesting a potential antiarrhythmic effect beyond metabolic and hemodynamic improvements [78].

In conclusion, early concerns regarding a potential pro-arrhythmic effect of GLP-1 RAs were primarily derived from limited observational data and have not been substantiated by subsequent large cardiovascular outcome trials or meta-analyses, which overall suggest a neutral or potentially favorable arrhythmic profile.

#### 4.2.3. Sodium Glucose Cotransporter-2 Inhibitors (SGLT-2i)

SGLT2 inhibitors (SGLT-2i) were originally developed as glucose-lowering agents and remain a central component of the pharmacologic management of DM [79]. Beyond their antihyperglycemic properties, extensive evidence from large multicenter randomized trials has demonstrated that SGLT-2i provide substantial cardiovascular and renal protection. These benefits include significant reductions in hospitalization for heart failure, cardiovascular mortality, all-cause mortality, and progression of chronic kidney disease, both diabetic and non-diabetic patients with elevated cardiovascular or renal risk [80,81]. Notably, these effects occur largely independent of glycemic control, supporting the presence of intrinsic cardioprotective mechanisms [79].

SGLT-2i act by blocking renal glucose reabsorption, thereby enhancing glucosuria and inducing mild natriuresis; moreover, they have been reported to reduce plasma volume and to exert other beneficial effects [82,83,84,85].

SGLT-2i have been evaluated in large multicenter randomized trials, which consistently demonstrated their efficacy in reducing cardiovascular mortality, hospitalizations for worsening heart failure, and adverse renal outcomes [82,86].

Although their potential antiarrhythmic properties remain incompletely elucidated, several mechanistic pathways have been proposed. SGLT-2i inhibit the myocardial sodium–hydrogen exchanger, lower intracellular sodium and calcium concentrations, and reduce atrial stretch through improved volume regulation—processes associated with attenuation of myocardial hypertrophy, fibrosis, and with adverse structural remodeling [86]. Additionally, accumulating evidence suggests that epicardial adipose tissue contributes to AF pathogenesis [87]. In a study of 40 patients with DM and coronary artery disease, Sato et al. demonstrated that Dapagliflozin reduced epicardial adipose tissue volume on computed tomography, supporting a possible metabolic and structural mechanism for AF prevention [88].

Clinical trial data assessing AF outcomes remain heterogeneous. A recent meta-analysis by Zheng et al. including 20 RCTs and 63,604 participants reported that SGLT-2i therapy was associated with a reduction in incident AF among patients with type 2 DM, HF, and chronic kidney disease [86].

In the DECLARE–TIMI 58 trial, Dapagliflozin was associated with the lowest incidence of AF events, yet without an associated decrease in stroke risk in patients with or without type 2 DM [86]. Additional trials are currently exploring the broader antiarrhythmic effects of SGLT-2i beyond AF [88].

However, the current evidence is inconsistent, with heterogenous results. A large recent meta-analysis of 46 randomized controlled trials involving over 100,000 individuals found no statistically significant reduction in AF incidence with SGLT-2i across populations with DM, heart failure, chronic kidney disease, or cardiometabolic risk, although a non-significant trend favored SGLT-2i [81].

Complementary evidence from observational research further complicates interpretation. A meta-analysis of six large real-world studies including 847,028 patients with type 2 DM showed that SGLT-2i were associated with a significantly lower risk of new-onset AF compared with GLP-1 receptor agonists (RR 0.76; 95% CI 0.65–0.89), without differences in stroke risk. Meta-regression suggested that male sex might weaken this protective effect [89].

Taken together, existing data suggest a possible but still incompletely defined antiarrhythmic effect of SGLT-2i. Given the discordant findings across RCTs, meta-analyses, and pharmacological classes, rigorously designed prospective randomized trials are required to identify the true magnitude of AF risk reduction and to determine whether SGLT-2i exert clinically meaningful antiarrhythmic effects [35,88,90].

These findings underscore the importance of conducting mechanistic and clinical studies to clarify the pathways behind these potential benefits and to address the inconsistencies present in current evidence [91].

### 4.3. Cardioversion of Atrial Fibrillation and Glucose-Lowering Therapies

Multiple studies have indicated that DM may reduce the success of cardioversion in AF. Case–control and multicenter cohort studies have shown that patients with DM experience lower rates of immediate cardioversion and sinus rhythm maintenance, with glycemic control emerging as an independent predictor of treatment failure [92,93]. Moreover, DM has been associated with a higher likelihood of early AF recurrence following cardioversion or ablation [94].

Recent evidence suggests that SGLT-2i may exert direct antiarrhythmic effects in the atria, potentially improving outcomes in patients with DM undergoing cardioversion. Experimental studies demonstrated that acute treatment with Dapagliflozin reduces atrial excitability by lowering the amplitude and upstroke velocity of action potentials in isolated atrial cardiomyocytes. These effects were more pronounced in atrial than in ventricular cells and were associated with a significant decrease in peak sodium current, alongside moderate inhibition of transient outward potassium currents. Translational studies in large animal models confirmed that dapagliflozin acutely slows atrial conduction, an effect that could facilitate both the acute cardioversion of paroxysmal AF and the rhythm control of persistent AF. Together, these findings indicate that beyond glycemic control, SGLT-2i might directly modulate atrial electrophysiology, offering a potential therapeutic advantage for patients with diabetes at risk of atrial arrhythmias [95].

### 4.4. Catheter Ablation of Atrial Fibrillation and Glucose-Lowering Therapies

Catheter ablation is a well-established strategy for managing symptomatic AF in patients who do not respond adequately to antiarrhythmic drugs. In individuals with DM, ablation has demonstrated clinical benefits, including better rhythm control, enhanced quality of life, and reduced rates of hospitalization [96,97]. The impact of DM on AF recurrence after ablation remains a matter of debate. A meta-analysis of 15 studies involving 1464 diabetic patients indicated that higher baseline HbA1c levels were linked to an increased risk of AF recurrence, highlighting the potential importance of optimal glycemic control for improving post-ablation outcomes [98].

#### 4.4.1. GLP-1 RAs

The influence of GLP-1 RA on AF recurrence after AF ablation remains uncertain, with available data yielding mixed results. A large multicenter observational analysis evaluating GLP-1 RA use within the year before catheter ablation (in a population that also included patients with DM) found no association between GLP-1 RA therapy and subsequent AF recurrence, need for another ablation procedure, or initiation of antiarrhythmic medication, nor any differences in major clinical endpoints over one year of follow-up [99].

In contrast, a prospective cohort study of patients undergoing first-time AF ablation reported that Semaglutide therapy was linked to a significantly lower risk of AF recurrence at 12 months (HR 0.68), suggesting that GLP-1 RAs may offer rhythm-modulating benefits beyond metabolic effects [98].

Overall, these findings indicate that despite preliminary data of anti-arrhythmic benefit, the overall evidence remains inconsistent, emphasizing the need for adequately powered randomized trials to clarify the post-ablation role of GLP-1 RAs for rhythm management.

#### 4.4.2. SGLT-2i

Recent evidence suggests that SGLT-2i may confer electrophysiological and hemodynamic benefits in patients with persistent AF and heart failure, even in the absence of type 2 DM [99]. In a prospective study, in heart failure patients without DM undergoing catheter ablation, the periprocedural SGLT-2i therapy was associated with significantly lower left atrial pressures and reduced NT-proBNP levels at the time of ablation and during early follow-up. These hemodynamic improvements were associated with higher rates of arrhythmia-free survival, both in the early post-procedural period and at one-year follow-up, compared with patients who did not receive SGLT-2i therapy. The data suggest that SGLT-2i may favorably modulate atrial electrophysiology and mitigate atrial stretch, thereby enhancing the efficacy of AF catheter ablation. These results suggest that SGLT-2i may exert pleiotropic antiarrhythmic benefits beyond their effects on glycemic control [99].

Taken together, the evidence linking glucose-lowering therapies to AF risk varies substantially across drug classes and study designs. Observational and post hoc analyses dominate the available literature, particularly for metformin, sulfonylureas, and thiazolidinediones, whereas randomized cardiovascular outcome trials provide more robust but still indirect data for GLP-1 RAs and SGLT2i. While some agents appear to be associated with a lower incidence of AF, these findings should be interpreted cautiously, as differences in patient selection, background cardiovascular risk, and glycemic control may partly explain the observed associations. From a clinical standpoint, no glucose-lowering therapy can currently be recommended primarily for AF prevention, although certain drug classes may offer indirect atrial benefits through improvements in metabolic, hemodynamic, and inflammatory profiles (Table 1).

## 5. Conclusions

DM is strongly associated with an increased incidence and prevalence of AF through a complex interplay of metabolic, inflammatory, structural, and electrophysiological alterations. Rather than acting as isolated mechanisms, these processes converge to promote a diabetes-related atrial myopathic phenotype, characterized by progressive atrial remodeling, electrical instability, and heightened arrhythmogenic vulnerability.

Although several glucose-lowering therapies have been associated with a lower incidence of AF in observational studies and post hoc analyses, the current evidence remains heterogeneous and largely non-randomized. Metformin appears neutral or slightly protective. Among newer therapies, several observational studies and post hoc analyses of randomized trials suggest that SGLT2i may be associated with a lower incidence of AF. GLP-1 RAs, despite substantial benefits on stroke and major cardiovascular events, have not demonstrated a clear reduction in new-onset AF.

From a clinical perspective, AF in patients with DM should be viewed as a manifestation of an underlying atrial cardiomyopathy rather than an isolated rhythm disorder, underscoring the importance of comprehensive cardiovascular risk factor management. Future research should focus on prospective, adequately powered randomized trials with AF as a predefined endpoint, as well as mechanistic studies aimed at identifying patient subgroups most likely to derive atrial benefit from targeted metabolic and anti-inflammatory interventions.

## Figures and Tables

**Table 1:** Comparative effects of antidiabetic drug classes on atrial fibrillation: clinical evidence, mechanistic pathways, and strength of evidence.

Class of Medication	Actual Evidence	Mechanistic Pathways	Comparation with Other Classes of Medication	Strength of Evidence
Metformin	associated with a lower risk of new-onset AF associated with fewer AF-related hospitalizations	activation of AMP-activated protein kinase (AMPK), which facilitates intracellular calcium handling	associated with a lower AF risk compared with sulfonylureas and insulin in observational studies	Observational studies
Thiazolidinedione	associated with a reduced risk of new-onset AF possible reduction in recurrent AF, mainly with pioglitazone	anti-inflammatory and antioxidant mechanism	associated with a lower AF incidence compared with other second-line therapies in selected cohorts	Observational studies
DPP-4i	overall neutral to modest effect on AF incidence	no influence	AF risk generally comparable to or higher than with SGLT2i in observational studies comparisons	Observational studies
SGLT-2i	associated with a lower incidence of AF in several observational studies inconsistent reduction in AF incidence across randomized trials potential benefit in selected clinical settings (e.g., cardioversion, post-ablation)	inhibit the myocardial sodium–hydrogen exchanger lower intracellular sodium and calcium concentrations reduce atrial stretch through improved volume regulation reduce epicardial adipose tissue volume direct antiarrhythmic effects	may be associated with a lower AF risk compared with DPP-4 inhibitors and GLP-1 receptor agonists in real-world studies	RCTs Observational studies Meta-analyses
GLP1-RAs	overall neutral effect on AF incidence possible reduction in AF recurrence in selected populations	anti-inflammatory and antioxidant mechanism reduce atrial fibrosis optimize cardiac metabolism	AF risk generally comparable to DPP-4i less consistent AF reduction compared with SGLT2i	Animal studies RCTs Observational studies Meta-analyses
Sulfonylureas	associated with a higher risk of AF in some observational studies inconsistent findings across cohorts	an effect on cardiac potassium channels indirectly through their risk of hypoglycemia, which triggers adrenergic activation	generally associated with a higher AF risk compared with metformin	Observational studies
Insulin	associated with a higher incidence of AF in observational studies findings likely influenced by disease severity and comorbidity burden	hypoglicemia glicemic variability long term diabete	no evidence of AF risk reduction	Observational studies

## Data Availability

The data supporting the findings of this study can be provided by the corresponding author upon reasonable request.
